# The Simulation of the Recharging Method Based on Solar Radiation for an Implantable Biosensor

**DOI:** 10.3390/s16091468

**Published:** 2016-09-10

**Authors:** Yun Li, Yong Song, Xianyue Kong, Maoyuan Li, Yufei Zhao, Qun Hao, Tianxin Gao

**Affiliations:** 1 School of Opto-Electronics, Beijing Institute of Technology, Beijing Key Laboratory for Precision Optoelectronic Measurement Instrument and Technology, Beijing 100081, China; 2120150589@bit.edu.cn (Y.L.); 3120120280@bit.edu.cn (X.K.); 2932183@bit.edu.cn (M.L.); zhaoyufei@bit.edu.cn (Y.Z.); qhao@bit.edu.cn (Q.H.); 2 School of Life Science, Beijing Institute of Technology, Beijing 100081, China; gtx@bit.edu.cn

**Keywords:** implantable biosensors, solar radiation, sunlight, Monte Carlo method

## Abstract

A method of recharging implantable biosensors based on solar radiation is proposed. Firstly, the models of the proposed method are developed. Secondly, the recharging processes based on solar radiation are simulated using Monte Carlo (MC) method and the energy distributions of sunlight within the different layers of human skin have been achieved and discussed. Finally, the simulation results are verified experimentally, which indicates that the proposed method will contribute to achieve a low-cost, convenient and safe method for recharging implantable biosensors.

## 1. Introduction

Recently, more and more implantable biosensors have been used in the medical field. As a result, medical monitoring and treatment can be achieved directly within the human body [1]. However, current battery technologies can only provide operation times of 2–5 years [2]. Consequently, the in vivo recharging method of implantable biosensors has become a crucial issue, which has attracted a great deal of research interests [3,4].

Although several recharging methods of implantable biosensors have been researched in recent years [5,6], it is still proving difficult to meet its requirements of high energy efficiency, stability and convenience. For instance, the radio frequency electromagnetic induction method [7,8] is not likely to produce enough ambient radio frequency energy for wirelessly powering miniature biosensors [9]. Besides, the heat produced in the process of electromagnetic induction interferes with the physiological activity of the human body [10]. Although the recharging method based on low-frequency magnetic fields is considered as a reliable method and has been used in a medical field, the devices with large dimension used in this method hinder the implantation as well as the application of implantable biosensors [11]. Compared with the methods mentioned above, the photovoltaic recharging method offers the advantages of safety, high energy efficiency and stability [12], which is considered as a promising recharging method of implantable biosensors. However, in the current photovoltaic recharging methods, a laser source is generally used to achieve the required power density in tissue, which may do harm to the human body as its high power can cause skin damage [13] as well as increase the size and cost of recharging devices.

On the other hand, if sunlight can be used for recharging implantable biosensors, it will lead to a recharging method of implantable biosensors with the characteristics of low cost, convenience, and safety. The comparison between the recharging method based on solar radiation and other methods is summarized in Table 1.

As for recharging implantable biosensors using sunlight, the spectrum and irradiance distribution of the sunlight on the surface of human skin should be analyzed. Moreover, sunlight is a multi-wavelength light, the propagation of which is difficult to simulate within different layers of skin tissue. As a result, the energy distribution of sunlight within human skin remains undetermined so far. In this paper, a method for recharging implantable biosensors based on solar radiation is proposed. Firstly, we developed the models of the proposed method, which include the models of sunlight source, wearable optical system, and skin tissue. Secondly, the recharging processes have been simulated using MC method, while the energy distributions of sunlight within the different layers of human skin have been achieved and discussed. Finally, the simulation results are verified experimentally. Our results indicate that sunlight could serve as a safe, stable, and low-cost energy source for implantable biosensors.

## 2. Modeling

### 2.1. Method

**Figure 1:**
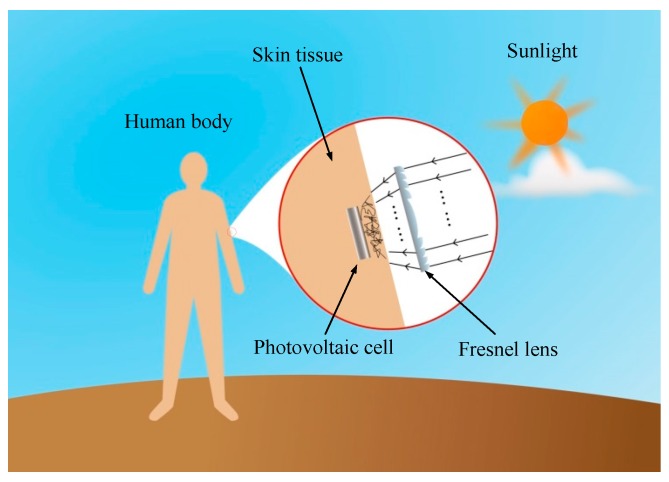
The recharging method of implantable biosensors using sunlight.

In the recharging method of implantable biosensors based on solar radiation, as shown in Figure 1, firstly, the multi-wavelength sunlight is focused by a Fresnel lens. Then, the focused light arrives at the skin surface, and transmits within the skin tissue. Finally, light that arrives at the implantable photovoltaic cell is converted into electric energy. Ultimately, the recharging of implantable biosensors based on solar radiation is achieved using sunlight.

### 2.2. Solar Radiation Model

#### 2.2.1. Spectral Range

**Figure 2:**
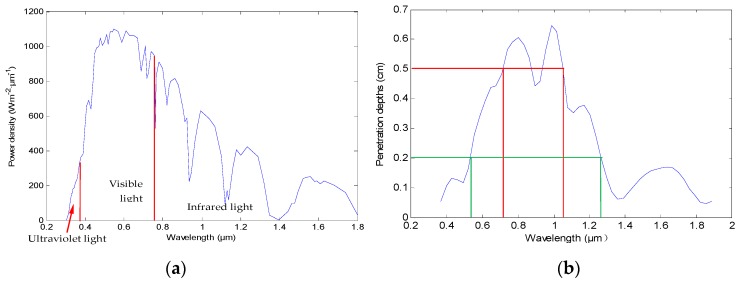
(**a**) The curve of power density of the earth surface; (**b**) The penetration depths in skin tissue of the light corresponding to 0.40–1.80 μm.

Generally, the energy distribution of the earth surface is represented by the power density, which is the ratio of solar radiation flux and the corresponding area. Figure 2a shows the sunlight power density of the earth surface corresponding to 0.3–1.8 μm [17]. On the other hand, the penetration depth of sunlight with the different wavelengths in the skin tissue should also be considered. Figure 2b shows the penetration depths in skin tissue of the light corresponding to 0.40–1.80 μm [18,19]. In the proposed method, silicon photovoltaic cell with high sensitivity in the spectral range of 0.70–1.00 μm [20] is used for photoelectric conversion. Meanwhile, ultraviolet light (0.30–0.38 μm) of the solar radiation, which may do harm to human tissue, is filtered using a filter. Finally, considering the factors of the spectral distribution of power density, the penetration depth of sunlight in skin tissue, and the high sensitivity spectral range of photovoltaic cell, 0.50–1.00 μm was chosen as the wavelength range in the proposed recharging method. Additionally, since this range is within the spectral range of lighting sources, as a result, even if the users stay indoors, a certain amount of energy can be obtained from lighting sources.

#### 2.2.2. Calculation Method

To determine the solar energy of skin surface of the human body, the solar energy of the earth surface should be calculated. Here it is assumed that *r*_0_ represents the ratio of solar irradiation per hour (*I*) and solar irradiation per day (*H*_a_) of the solar energy on the earth surface. According to the latitude, climate, and the other conditions of the location, *H*_a_ can be determined using the following equation.

(1) $$H_{a}=H_{0}(a+b\cdot \frac{\bar{n}}{N})$$

where *H*_0_ is the average irradiation per day of the exoatmosphere area which is corresponding to the earth surface where the human body is situated. $\bar{n}$ is the average solar radiation time per day, *N* is the longest solar radiation times of a day, and *a* and *b* are the climate constants, which are related to climate and geographic position. Finally, *r*_0_ can be calculated according to the following equations [21,22].

(2) $$\left\{ \begin{array}{l} r_{0}=\frac{\pi }{24}(a+b\cos\omega )\frac{\cos\omega -\cos\omega_{r}}{\sin\omega_{r}-\frac{\pi \omega_{r}\cos\omega_{r}}{180}}\\ \omega_{r}=\mathrm{arc}\;\cos(-\tan\varphi \cdot \tan\delta )\\ a=0.409+0.5016\;\sin(\omega_{r}-60)\\ b=0.6609-0.4767\;\sin(\omega_{r}-60)\\ \delta =23.45^{\circ }\sin(360\frac{284+n}{365}) \end{array}\right.$$

where *ω* is the hour angle, *ω_r_* is the sunrise hour angle, *ϕ* is the local geographic latitude, *δ* is solar declination, *n* is the number of the days calculated from January 1 of a year. Finally, according to *H*_a_ and *r*_0_, the solar irradiation per hour (*I*) of the skin surface can be determined using *I* = *H*_a_ × *r*_0_.

### 2.3. Fresnel Lens Model

**Figure 3:**
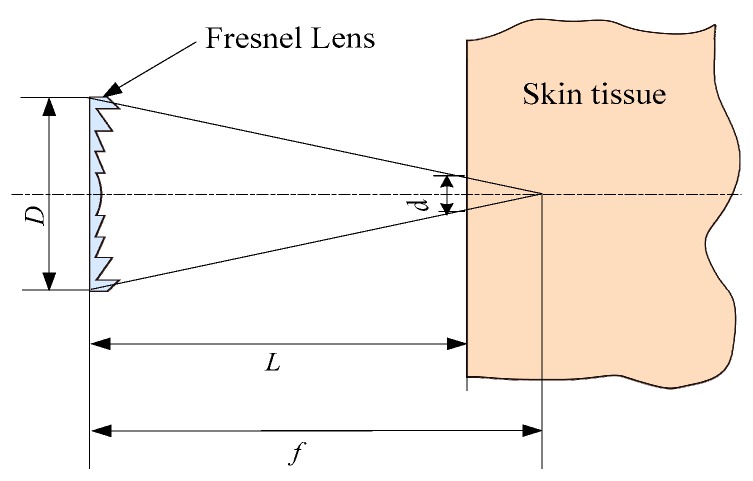
Parameters of Fresnel lens.

Generally, the energy density of solar radiation is relatively low in the morning, evening, and on a cloudy day. To provide enough energy for the implantable biosensors, the solar radiation is gathered through a Fresnel lens in the proposed method. Moreover, the application of Fresnel lens also can decrease the size of a sunlight spot, which is helpful to reduce the photosensitive surface of the implantable cell. Additionally, a Fresnel lens with flat form is suitable for the wearable applications. Figure 3 shows the parameters of the Fresnel lens, in which *D* represents the aperture, *d* is the spot diameter on the skin surface, *f* is the focal length, and *L* is the distance between skin and Fresnel lens. According to the geometrical optics theory, the spot area (*S_r_*) of sunlight on the skin surface can be expressed by Equation (3). Additionally, the use of a Fresnel lens does not introduce a safety issue, which is discussed in Section 3.1.4.

(3) $$S_{r}=\pi \cdot {\left(\frac{d}{2}\right)}^{2}=\frac{1}{4}\pi {\left[\frac{(f-L)D}{f}\right]}^{2}$$

### 2.4. Skin Tissue Model

**Figure 4:**
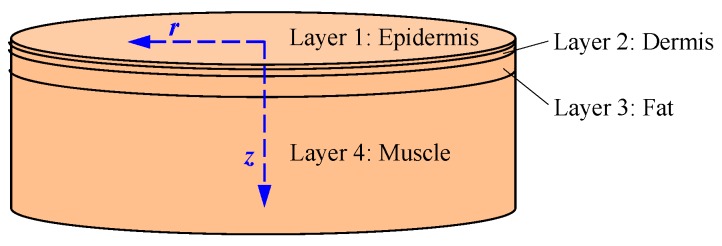
Model of the skin tissue of human body.

The skin tissue of human body was modeled geometrically by four infinitely wide layers, which include epidermis, dermis, fat, and muscle as shown in Figure 4. The thicknesses of the epidermis, dermis, and fat layers are 0.0065 cm, 0.125 cm, and 1.2 cm, respectively [23,24]. Meanwhile, the thickness of muscle is set to infinity because sunlight generally cannot penetrate the muscle layer. Moreover, in the cylindrical coordinates of the developed model shown in Figure 4, the *r* axis plane is parallel to the tissue surfaces, while the *z*-axis is perpendicular to the skin surfaces.

## 3. Simulations

### 3.1. Energy Distribution of Sunlight on the Skin Surface

#### 3.1.1. Simulation Condition

In order to determine the spot position of solar radiation through the Fresnel lens on skin surface, the simulations corresponding to the vertical incidence and oblique incidence of sunlight were carried out. The simulation conditions include: the time is at noon in summer solstice (according to the method of Section 2.2.2, the calculated value of the incident solar power is 0.480 W), *d* = 2.00 cm, *D* = 3.00 cm, *L* = 0.20 cm, and *f* = 1.60 cm.

#### 3.1.2. Vertical Incidence

**Figure 5:**
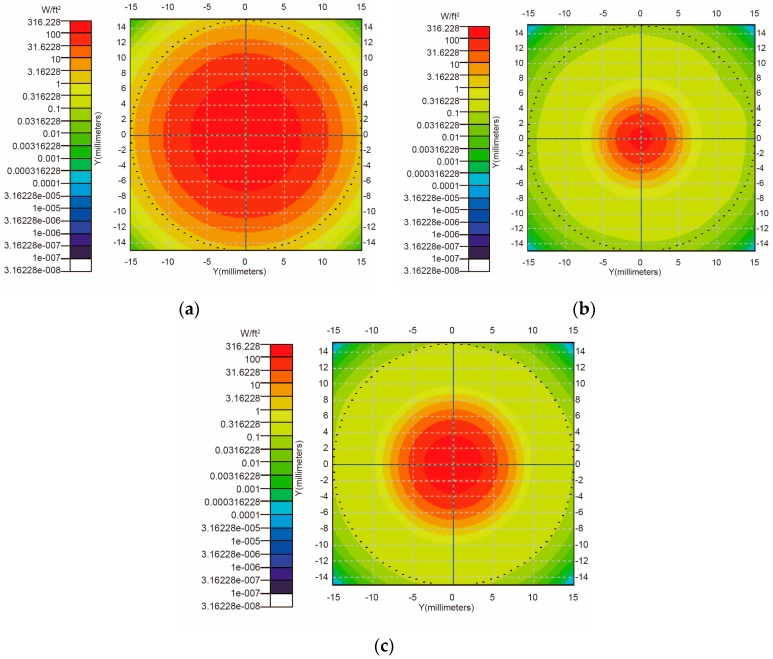
Luminous flux distributions of sunlight on the skin surface, corresponding to the cases that *L* are 0.53 cm (**a**); 1.60 cm (**b**); and 2.10 cm (**c**); respectively.

The transmissions of sunlight in the air and Fresnel lens were simulated by using TracePro software. According to Equation (3), if *d* = 2.00 cm, *L* = 0.53 cm. Figure 5 shows the vertical incidence results corresponding to the cases that *L* = 0.53 cm as well as *L* = *f* = 1.60 cm and *L* = 2.10 cm (*f* + 0.50 cm). According to Figure 5, the results corresponding to the cases that *L* = 0.53 cm has a comparatively big spot area with high luminous flux, which is helpful to achieve more electric energy. Therefore, in our simulations, *L* is set as 0.53 cm.

#### 3.1.3. Oblique Incidence

Generally, sunlight arrives at the skin surface of the human body with oblique incidence. In these cases, the spot center on the skin surface is NOT on the optical axis of Fresnel lens. To determine the relative position between the Fresnel lens and the implantable photovoltaic cell in the plane vertical to the optical axis, the corresponding simulations were also carried out. The parameters correspond to 10:00, 12:00, and 14:00 of summer solstice of solar radiation are shown in Table 2 [25]. 

**Figure 6:**
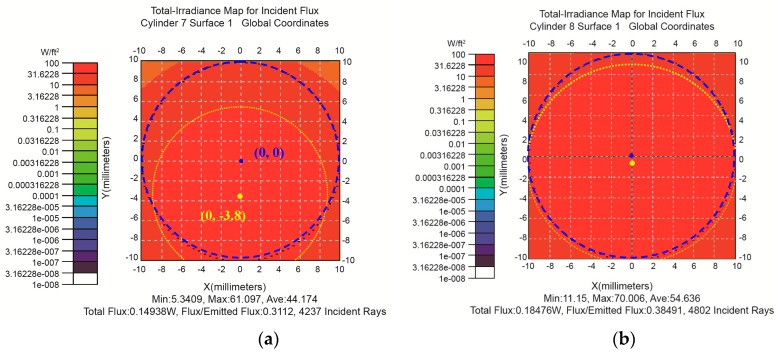
Luminous fluxes distribution corresponding to the case that the optical axis of Fresnel lens was at the center of implantable photovoltaic cell (**a**); and was shifted 0.50 cm of the implantable photovoltaic cell (**b**) at the times of 12:00.

According to the altitude angle, azimuth angle and incident energy in different times, the energy distribution of the skin surface after the Fresnel lens can be obtained, as shown in Table 2. Figure 6a shows the energy distribution corresponding to 12:00, in which the point (0, −3.8) is the center of luminous fluxes distribution on skin surface, while the point (0, 0) represents the center position of implantable photovoltaic cell in the plane vertical to the optical axis. It can be found from Figure 6a that the center of luminous fluxes distributions is generally located at the lower of the center position of implantable photovoltaic cell. Therefore, in order to achieve more electric energy, the optical axis of the Fresnel lens was shifted by 0.50 cm in our following simulations. Therefore, it was close to the center of implantable photovoltaic cell, as shown in Figure 6b. As a result, the energies corresponding to 10:00, 12:00 and 14:00 were increased to 1.6 times, 1.4 times and 1.7 times of the previous energies, as shown in Table 2.

#### 3.1.4. Safety

Safety is the most important issue for all the recharging methods for implantable medical devices. According to the regulation issued by International Commission on Non-Ionizing Radiation Protection (ICNIRP), the radiation intensity corresponding to visible light and near infrared light (400–1400 nm) on human skin should less than 2.0 × 10^3^ C_A_·W/m^2^ (C_A_ ≥ 1) [26]. In the results shown in Table 2, the maximum solar energy of the spot with 1.00 cm radius on skin surface is 0.1848 W, which means that the radiation intensity is result is 588.53 W/m^2^. Therefore, the radiation intensity on skin surface of the proposed method is less than 2.0 × 10^3^ W/m^2^.

### 3.2. Energy Distributions within Skin Tissue

#### 3.2.1. Method

In this paper, we proposed a simulation method of sunlight transmission based on energy superposition, which can be described as follows: (1) The energy distributions corresponding to each wavelength of the sunlight are achieved by using the simulations based on MC method; (2) According to the spectral distribution of solar radiation, the weight of each wavelength is determined; (3) According to the luminous flux and weight of the sunlight corresponding to each wavelength, the energy distribution within skin tissue is determined using the method of weighted averaging.

#### 3.2.2. Energy Distribution along *r* Axis

**Figure 7:**
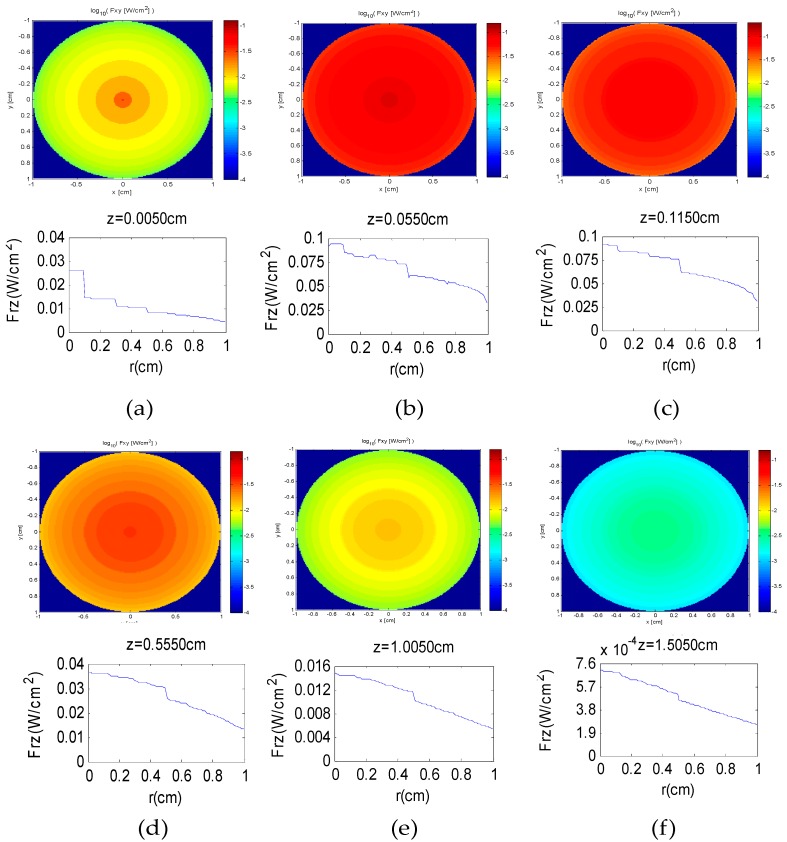
Pseudo-color charts and curve charts of luminous flux along the *r* axis corresponding to the different depths along the *z* axis, which include 0.0050 cm (**a**); 0.0550 cm (**b**); 0.1150 cm (**c**); 0.5550 cm (**d**); 1.0050 cm (**e**) and 1.5050 cm (**f**); respectively.

The transmission of sunlight within four tissue layers (epidermis, dermis, fat, and muscle) was simulated using the mentioned method. Figure 7 shows the Pseudo-color charts and curve charts of luminous flux along *r* axis corresponding to the different distances of the skin tissue along *z* axis, which include 0.0050 cm (a); 0.0550 cm (b); 0.1150 cm (c); 0.5550 cm (d); 1.0050 cm (e) and 1.5050 cm (f), respectively. According to Figure 7, the luminous flux gradually decreases from the center to the edge along the *r* axis. On the other hand, as *z* increases from 0.0050 cm to 0.0550 cm, the whole luminous flux of the spot increases gradually from 0.0912 W to 0.1813 W, and decreases gradually from 0.1813 W to 0.0065 W when *z* increases from 0.0550 cm to 1.5050 cm.

#### 3.2.3. Energy Distribution along *z* Axis

**Figure 8:**
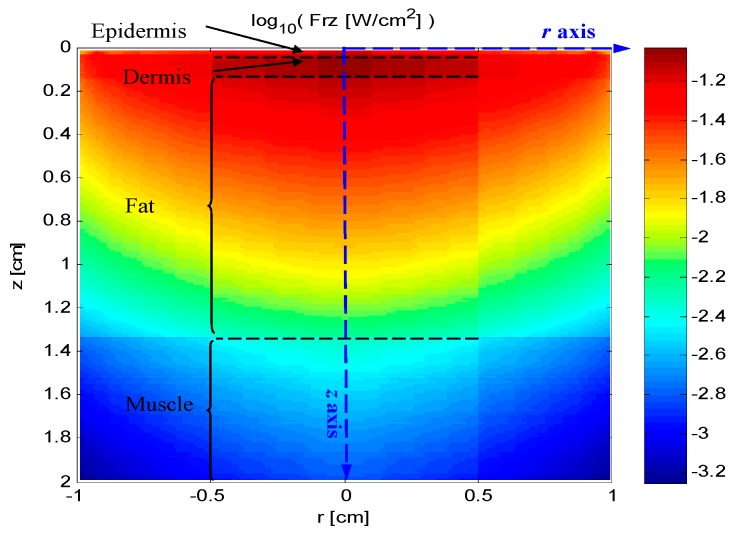
The longitudinal section of the energy distributions, of which horizontal axis represents *r* axis, and vertical axis represents *z* axis.

Figure 8 shows the longitudinal section of the energy distributions, of which horizontal axis represents *r* axis, and vertical axis represents *z* axis with the depth of 2.00 cm. According to Figure 8, the luminous fluxes are symmetric along the *z* axis. At the same time, the comparatively high luminous fluxes mainly focus on the symmetry area with 0.50 cm radius and 1.00 cm depth. Moreover, the luminous fluxes gradually decrease from 0.077 W/cm^2^ to 0.015 W/cm^2^ as the depth increases from 0.10 cm to 1.00 cm. Additionally, the energy (*E*) at the depth of *D* with the radius of *R* can be calculated by Equation (4).

(4) $$E=\sum_{d=0}^{D/D_{s}}\sum_{r=0}^{R/R_{s}}f(R_{s}\cdot r,D_{s}\cdot d)\cdot S_{r}$$

where *R_s_* is the step length of radius, *D_s_* is the step length of depth, *S_r_* is the area corresponding to the different radiuses and *f* is the simulation results of luminous flux corresponding to the different radiuses and depths. As a result, the calculation value of the energy (*E*) at the depth of 1.00 cm along *z* axis is 0.028 W under the conditions that *D* = 1 cm, *R* = 1 cm, *R_s_* = 0.01 cm and *D_s_* = 0.01 cm. Because the efficiencies of photovoltaic cells are generally within the range of 10%–20% [6], 0.028–0.056 W electric power can be achieved using the proposed method. According to this result and the previous work [11], which indicated that the photovoltaic cell charged by a power density of 22 mW/cm^2^ for 17 min can support for a pacemaker to operate for 24 h, the photovoltaic cell charged by solar radiation for one hour can support the normal work of a pacemaker for more than two days. Due to the fact that the proposed method can be used during the whole daytime, the power deposited by solar radiation is sufficient for implantable biosensors.

## 4. Physical Experiments

### 4.1. Experimental Device

**Figure 9:**
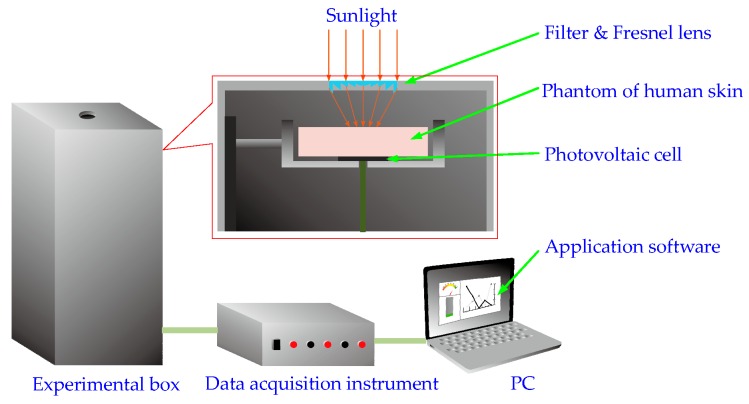
Experimental device of the recharging method of implantable biosensors based on solar radiation.

Our experimental device is shown in Figure 9, which consists of a filter (bandwidth: 0.50–1.00 μm), a Fresnel lens (*D* = 3.00 cm, *f* = 1.60 cm), a photovoltaic cell (photoelectric conversion efficiency: 16%, photosensitive surface: 2.00 cm × 2.00 cm, spectral response range: 0.40–1.10 μm, operating current: <200 mA and short circuit current: <±0.5%), a data acquisition instrument (sampling precision: 12 bit), a PC with the data storage and display application software (LabView, National Instruments, Austin, TX, USA). In this experiment, the photovoltaic cell is directly connected to the data acquisition instrument (in practical application of the proposed method, a charging circuit will be needed). Because pig’s skin and human skin are similar in both physiological characteristics and physiological structure [27], pork is suitable for the phantoms of human body. On the other hand, the layer thickness of pork skin is different from that of human skin. Moreover, it is generally difficult to measure the thickness accurately. Therefore, only the fat tissues of pork with different thicknesses (0.1 cm, 0.2 cm, 0.4 cm, 0.6 cm, 0.8 cm and 1.0 cm) were used in our experiments.

### 4.2. Results and Discussions

**Figure 10:**
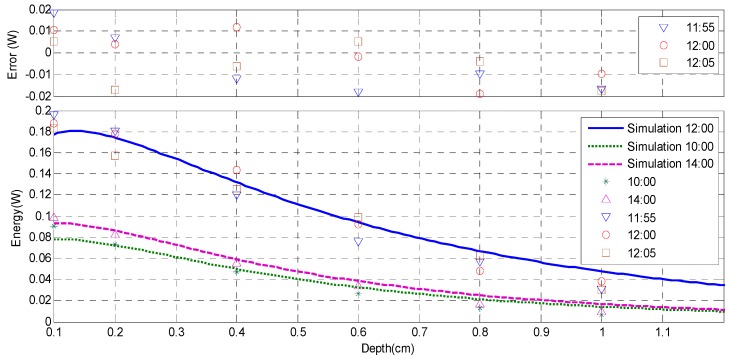
The physical measurement results and the corresponding simulation results of the recharging implantable biosensors based on solar radiation.

In order to verify the proposed method, the physical experiments of the recharging implantable biosensors based on solar radiation were carried out at 10:00, 12:00 and 14:00 of a day near summer solstice in Beijing, China, while the solar zenith angle is 73.53°, the solar azimuth is 0° [28] and the energy of solar radiation on the surface of the earth is 0.0679 W/cm^2^. Meanwhile, the corresponding simulations were also carried out by using the same parameters setting with the physical experiments, and the energies of the different depths were calculated using the method described in Section 3.2.3. Additionally, due to the fact that the optical characteristics of pork will change after 24 h [29], physical experiment was not carried out in more than one day. Figure 10 shows the physical experimental results and corresponding simulation results, of which the horizontal axis represents the thickness of the fat tissues, while vertical axis represents the achieved energy. In order to compare with the simulation results, which are the energies before the photoelectric conversion of the photovoltaic cell, the measurement results shown in Figure 10 are the results of the experimental device divided by the photoelectric conversion efficiency of the photovoltaic cell. In Figure 10, the experimental result of 12:00 is represented as the results corresponding to the times of 11:55, 12:00 and 12:05, respectively. Meanwhile, an error bar which shows the differences between the three experimental results and the simulation result is also provided. 

According to Figure 10, the simulation results basically agree with the physical measurement results, while the average error between the simulation results of 12:00 and the average value of the corresponding three experimental results is only 0.008 W. Meanwhile, both the simulation results and the measurement results decrease with the increase of the thickness of fat tissue. Concretely, the energy of the measurement result decreases from 0.189 W to 0.033 W as the thickness increases from 0.10 cm to 1.00 cm, which indicates that approximately 0.033 W energy can be achieved at the depth of 1.00 cm of fat tissue at the time of 12:00. On the other hand, compared with the results of 10:00 and 14:00, the results of 12:00 have the maximum energies. Meanwhile, the results of 14:00 are bigger than that of 10:00 at the depth range of 0.1–0.6 cm, and they become much closer to equal at the depth range of 0.7–1.0 cm. Additionally, it can be found from Figure 10 that the simulation results diverge from the experimental results at the beginning and ends of the depth range, which can be explained of following reasons. (1) The initial energies of skin surface shown in Figure 10 were the calculation results of the luminous flux distributions according to the simulation results achieved by using TracePro software, rather than the practical energies of sunlight on human skin. Therefore, it caused the comparatively big difference between simulation and experiment at the beginning of the depth range; (2) In the simulations based on MC method, it was assumed that the optical parameters of fat tissues keep constant. Actually, these parameters have a little variation with the increase of depth. Therefore, the difference between simulation and experiment becomes bigger as photons transmit deeper, especially at the ends of the depth range.

## 5. Conclusions

A method for recharging implantable biosensors based on solar radiation has been simulated and verified in this paper. Some conclusions can be reached, as follows: (1) In order to achieve more electric energy, the center of implantable photovoltaic cell should be located below a certain distance (such as 0.5 cm) of the optical axis of Fresnel lens; (2) All of the four tissue layers (*z* increases from 0.0050 cm to 1.5050 cm) has the maximum luminous flux at the spot center, and the luminous flux decreases gradually with the increase of *r*; (3) The comparatively high luminous fluxes mainly focus on the symmetry area with the radius of 0.50 cm and the depth of 1.00 cm; (4) The simulation results using MC method basically agree with the physical measurement results. 

Finally, according to the above discussions, sunlight could serve as a safe, stabile, convenient and low-cost energy source used for recharging implantable biosensors, which will promote its applications in the future.

## Figures and Tables

**Table 1:** Comparison between the recharging method using sunlight and other methods.

Methods	Reliability	Cost	Size (cm^2^)	Received Power (mW)	References
Radio frequency electromagnetic induction method	Low	Low	Unknown	10	[14]
Thermo electric generator method	Low	High	1.0	0.001	[11,15]
Previous optical recharging method	Low	High	10	4.00	[11,16]
The proposed recharging method	High	Low	Unknown	2.8–5.6	

**Table 2:** The parameters and energies of solar radiation.

Time	Altitude Angle (°)	Azimuth Angle (°)	Incident Energy (W)	Energy on Skin without shift (W)	Energy on Skin after Shift (W)
10:00	60.01	66.59	0.347	0.0562	0.0892
12:00	73.5	0	0.480	0.1494	0.1848
14:00	60.31	−66.60	0.433	0.0624	0.1071
